# Regulatory Effects of Maternal Intake of Microbial-Derived Antioxidants on Colonization of Microbiota in Breastmilk and That of Intestinal Microbiota in Offspring

**DOI:** 10.3390/ani14172582

**Published:** 2024-09-05

**Authors:** Dangjin Wu, Ran An, Di Wang, Luoxin Jiang, Liu Huang, Tenghui Lu, Weina Xu, Jianxiong Xu, Jing Zhang

**Affiliations:** 1 Shanghai Key Laboratory of Veterinary and Biotechnology, School of Agriculture and Biology, Shanghai Jiao Tong University, Shanghai 200240, Chinaran.an@sjtu.edu.cn (R.A.); wendy.b@sjtu.edu.cn (D.W.); 15201775283@163.com (L.J.); huangliusrh@163.com (L.H.); 15821186079@163.com (T.L.); xuweina@sjtu.edu.cn (W.X.); jxxu1962@sjtu.edu.cn (J.X.); 2 USJ-Kong Hon Academy for Cellular Nutrition, University of Saint Joseph, Macao 999078, China

**Keywords:** maternal feed, antioxidants, breastmilk microbiota, offspring gut microbiota, rat

## Abstract

**Simple Summary:**

How to effectively alleviate oxidative stress in pregnant sows and weaning piglets is one of the key issues in the pig industry. Maternal feeding is a key determinant of the composition and activity of the intestinal microbiota in both the mother and the offspring, while the effect of the maternal intake of microbial-derived antioxidants (MA) thereof remains to be elucidated. In the current study, we used Sprague Dawley rats as a model to investigate the impact of maternal MA additive on the microbiota of breastmilk, the maternal ileum, and the intestinal microbiota of the offspring on the day of birth and ten days afterwards, via analysis of 16S rRNA gene sequences. We found that the impact of MA was more obvious on the microbiota of mature milk than on that of colostrum. In addition, MA additive did not significantly affect maternal ileal microbiota, but affected offsprings’ colonic microbiota significantly ten days after birth. Collectively, the current study underlines colonic microbiota as a key target affected by maternal MA additive in the offspring with the potential to relieve oxidative stress by enriching beneficial bacteria, as well as aiding in the development of maternal feed for improved health outcomes in both mothers and their offspring. This study can provide a theoretical basis for the development of antioxidant feed and the improvement of animal welfare in the pig industry and elsewhere.

**Abstract:**

In this study, sixteen Sprague Dawley (SD) female rats and eight SD male rats were co-housed to mate. Pregnant SD female rats were fed with a control diet or an MA diet. Breast milk, maternal ileum, and intestinal samples of the offspring were collected at the day of birth and ten days afterwards. The results showed that the impact of MA was more obvious on the microbiota of mature milk (*p* = 0.066) than on that of colostrum. In addition, MA additive did not significantly affect maternal ileal microbiota, but affected offsprings’ colonic microbiota significantly ten days after birth (*p* = 0.035). From the day of giving birth to ten days afterwards, in addition to the increase in microbial richness and diversity, at genus level, the dominant bacteria of breastmilk changed from *Pseudomonas veronii* to *Bacillus* and *Lactococcus*. Different from breastmilk microbiota, ten days after giving birth, the maternal ileal microbiota and the offsprings’ intestinal microbiota were dominated by *Lactobacillus*. Instead of ileal microbiota, offsprings’ colonic microbiota is a key action site of maternal MA additive. Therefore, the current findings have significant implications for the development of maternal feed aimed at modulating the intestinal microbiota of offspring, ultimately leading to improved health outcomes for both mothers and their offspring.

## 1. Introduction

Sows always suffer from systemic oxidative stress during gestation and lactation due to the extensive metabolic demands of the mother and fetus resulting in the increased production of reactive oxygen species (ROS), which will affect the reproductive performance of sows [1,2,3]. At the same time, the early weaning technology of piglets is an important feeding technology, which can greatly improve breeding efficiency in the intensive pig farming industry [4,5]. However, studies have found that the early weaning process is often accompanied by serious oxidative stress, which will affect the intestinal function of piglets and seriously hinder the nutrient digestion and absorption of piglets [6,7]. Therefore, how to effectively alleviate oxidative stress in pregnant sows and piglets is one of the key issues in the pig industry.

ROS are a group of highly reactive molecules which play an essential role in various physiological processes [8,9,10,11]. Excessive ROS production could lead to oxidative stress which is, in part, associated with the pathogenesis of several chronic diseases, including cardiovascular disease, cancer, and neurodegenerative disorders [12,13]. Recently, the importance of ROS and their role in cellular signaling and homeostasis has been increasingly recognized, and their modulation has become a promising target for the prevention and treatment of oxidative stress-related diseases.

Antioxidants are molecules that can scavenge and neutralize ROS, thereby protecting cells and tissues from oxidative damage [14]. While antioxidants can be obtained from dietary sources, recent research has shown that the gut microbiota can also produce and deliver microbial-derived antioxidants (MA) to the host, such as short-chain fatty acids (SCFAs) [15]. Our previous studies found that MA additive reversed high-fat diet-induced liver oxidative stress in maternal and offspring mice, suggesting MA as a functional component to improve maternal–fetal health [16,17]. The intestinal microbiota is a complex and dynamic ecosystem of microorganisms that colonizes the intestinal tract and plays a crucial role in maintaining host health and well-being [18]. The intestinal microbiota can interact with the host’s immune system, modulate nutrient metabolism, and protect against pathogen colonization [19]. Dysbiosis, or an imbalance in the composition and activity of the intestinal microbiota, has been suggested to contribute to the development of several diseases [20], such as obesity [21], hypertension [22], and frailty [23].

Breastmilk is the primary source of nutrients for new-born infants and also contains bioactive compounds that can modulate the colonization and activity of the intestinal microbiota [24]. Recent studies have suggested that breastmilk also contains antioxidants that can protect against oxidative stress and inflammation [25]. However, the sources and regulatory mechanisms of these antioxidants are not fully understood.

Maternal diet has been shown to be a key determinant of the composition and activity of the intestinal microbiota in both the mother and the offspring [26], as well as the microbiota of breastmilk, which in turn affects the intestinal microbiota of the offspring [27]. Moreover, maternal intake of dietary antioxidants has been shown to affect the oxidative status of the mother and the offspring, and to modulate the composition and activity of the intestinal microbiota [28].

The current study aims to investigate the effects of maternal intake of MA on the colonization of the microbiota in breastmilk and on that of the intestinal microbiota in the mother and the offspring. We hypothesize that maternal intake of MA can modulate the microbiota composition of the breastmilk in the mother and the intestinal microbiota in the mother and the offspring, thereby protecting against oxidative stress and promoting a healthy microbiome.

## 2. Materials and Methods

### 2.1. Microbial-Derived Antioxidant (MA)

MA (KB-120, Shanghai Jiang Han Biotechnology, Shanghai, China) was made from the fruits of sea buckthorn and Rosa roxburghii, which were fermented by beneficial bacteria such as *Bacillus subtilis*, *Lactobacillus*, and beer yeast through solid–liquid complex fermentation. MA was composed of 503 mg/kg Fe, 367 mg/kg Mn, 1.07 mg/kg Cu, 0.18 mg/kg Se, 194,000 U/100 g superoxide dismutase, 322 mg/100 g vitamin C, 908 μg/100 g vitamin E, 4.43% total isoflavones, 1.37% isoflavones, 886 mg/100 g glutathione, 82.4 mg/100 g total saponins, 4.21% total amino acids, and 0.146% taurine [17,29,30,31,32].

### 2.2. Animal Experiment and Sample Collection

Sixteen Sprague Dawley (SD) female rats and eight SD male rats (body weight 250–270 g) were co-housed to mate in a ratio of 2:1. They were housed together in the afternoon at 6 p.m., and the male and female rats were separated the next morning at 8 a.m., with vaginal plug detection used as an indication of pregnancy. After the female rats became pregnant, they were randomly divided into two groups, each consisting of eight rats. One group was given a control diet (Basal diet, Suzhou Shuangshi Laboratory Animal Feed Science Co., Ltd., Suzhou, China), while the other group was given a control diet with 2% MA (98% Basal diet + 2%MA, MA was provided by Jang Han Biotechnology Co. Ltd., Shanghai, China). The basic diet’s nutritional composition is shown in Table 1.

The pregnant rats were housed individually in a custom-made stainless steel and plastic box measuring 25 cm × 40 cm. The room temperature was maintained between 20–25 °C with a relative humidity of 50–60%, and there was a 12 h light–dark cycle. The rats were allowed free access to food and water. The experiment ended 10 days after the rats gave birth. The mother rats and their offspring were separated for 6 h before sampling. The start of pregnancy was considered to occur the day on which the vaginal plug was detected, and the day of delivery was considered the end of pregnancy.

The weight and feed intake of the female rats were recorded every two days from the first day of pregnancy until the day of sample collection. The litter size and primary litter weight of each female rat was recorded within 24 h of delivery.

On the day of giving birth (day 1) and ten days after birth (day 10), breastmilk and ileum samples were collected from four lactating rats randomly, while whole intestine samples were collected from four offspring born from the above-selected mother rats separately. The lactating rats were intraperitoneally injected with 5 units of oxytocin (Ningbo second hormone factory, Ningbo, China), and after 10 min were anesthetized with sodium pentobarbital (45 mg/kg; P3761, Sigma, USA). The chest and abdomen of rats were sprayed with 75% alcohol, and the hair around the chest and nipples was cleaned with a hair shaver. The breast roots were pulled upward repeatedly. Gently squeezing the teat, 200 μL milk was collected by a 500 μL sterile tube with a pipette [33]. After milking, the ileum samples were collected in sterile tubes. The offspring were given pentobarbital sodium (45 mg/kg, P3761; Sigma, USA) through intraperitoneal injections and the whole intestine samples were collected. Collected samples were stored at −80 °C until analysis.

### 2.3. DNA Isolation and Sequencing

Following the manufacturer’s instructions, total bacterial DNA was extracted from colonic contents and milk samples using Fast DNA SPIN kits (MP Biomedicals, Santa Ana, CA, USA). Obtained genomic DNA was then used as a template for PCR amplification with 338F (5′-ACTCCTACGGGAGGCAGCA-3′) as the forward and 806R (5′-GGACTACHVGGGTWTCTAAT-3′) as the reverse primer to amplify the 16S rRNA gene V3-V4 region [34]. The PCR matrix was composed of 5 μL of Q5 reaction buffer (5×), 5 μL of Q5 High-Fidelity GC buffer (5×), 0.25 μL of Q5 High-Fidelity DNA Polymerase (5 U/μL), 2 μL (2.5 mM) of dNTPs, 1 μL (10 uM) of each primer, 2 μL of DNA template, and 8.75 μL of nuclease-free water. The PCR program was as follows: initial denaturation at 98 °C for 2 min, followed by 25 cycles consisting of denaturation at 98 °C for 15 s, annealing at 55 °C for 30 s, and extension at 72 °C for 30 s, with a final extension of 5 min at 72 °C. Obtained PCR products were further purified with Agencourt AMPure Beads (Beckman Coulter, Indianapolis, IN, USA) and pooled in equal amounts for paired-end 2 × 300 bp sequencing using an Illlumina MiSeq platform with a MiSeq Reagent Kit v3. The sequencing was performed by Shanghai Personal Biotechnology Co., Ltd. (Shanghai, China).

### 2.4. Data Processing

To integrate the paired-end sequencing data, a sliding window approach was used to perform quality filtering on the FASTQ format paired-end sequences. The window size was set to 10 bp with a step size of 1 bp, starting from the first base position of the 5′ end. A window with an average base quality ≥ Q20 (i.e., average sequencing accuracy ≥ 99%) was required, and the sequence was truncated at the first window with an average quality value below Q20. The truncated sequence length was required to be ≥150 bp and no ambiguous bases (N) were allowed. Then, the FLASH software v1.2.7 was used to pair and connect the overlapping bases between Read 1 and Read 2 sequences that passed the quality filtering. The overlapping base length between Read 1 and Read 2 was required to be ≥10 bp, and base mismatches were not allowed. The connected sequences were identified and assigned to corresponding samples based on the barcode sequence.

### 2.5. Sequence Analysis

The Quantitative Insights Into Microbial Ecology2 (QIIME2) pipeline was employed to process the sequencing data, as previously described [35]. For each operational taxonomic unit’s (OTU) representative sequence, default parameters were used to compare the OTU representative sequence with the template sequence of the Greengenes database [36] to obtain taxonomic information for each OUT, and they were used to calculate phylogenetic diversity and Shannon index.

### 2.6. Statistical Analysis

The 16S rRNA gene sequences were normalized to relative abundance and used to calculate phylogenetic diversity and Shannon index. The normality of studied data was tested with the Shapiro–Wilk test and the Wilcoxon signed-rank test or *t*-test was used for not normally or normally distributed data. Significant differences between groups were tested by permutational multivariate analysis of variance (PERMANOVA) based on pairwise Bray–Curtis and Jaccard distances at OTU level and used principal coordinate analysis (PCoA) to visualize microbiota variation. Variation partitioning was assessed by fitting testing variables, i.e., sampling timepoint and sample type, to the compositional data, using the CCA (canonical correlation analysis, CCA) function in combination with a Monte Carlo permutation in the vegan package [37]. Obtained *p* values were corrected for multiple testing following the Benjamini–Hochberg procedure by the false discovery rate (FDR). Corrected *p* values < 0.05 were considered to indicate significant differences. All statistics were conducted in R (R4-4.2.3).

## 3. Results

### 3.1. The Production Performance of Female Rats

As shown in Table 2, compared with the control diet-supplemented group, the average weight and average feed intake were significantly reduced during the pregnancy and lactation of female rats in the MA-supplemented group. However, there were no significant differences in litter size and primary litter weight between the two groups.

### 3.2. Sampling Timepoint and Sample Type Affect Microbiota Profile

**Figure 1:**
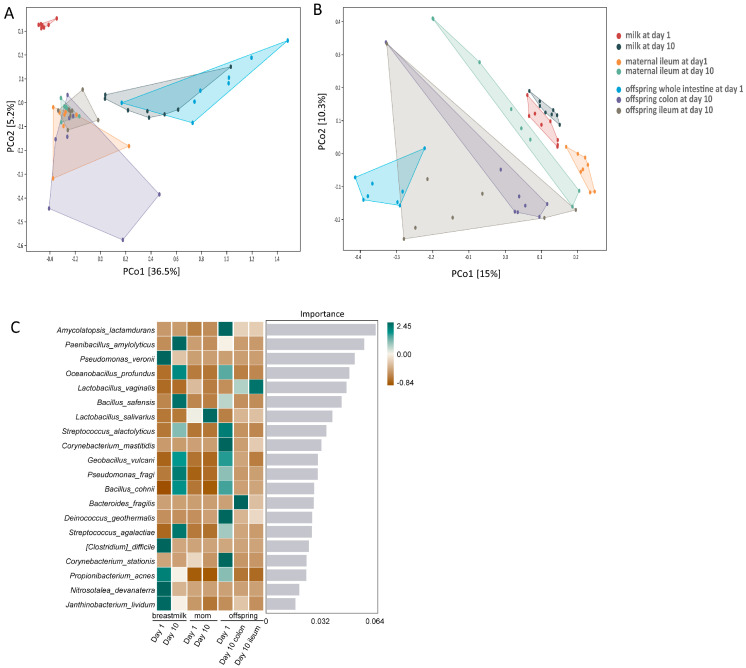
PCoA of microbial composition based on (**A**) weighted or (**B**) unweighted UniFrac distance matrices. Significant differences between samples based on pairwise weighted and unweighted UniFrac distances at ASV level were tested by PERMANOVA. The percentage of variation in microbial composition explained by the two principal coordinates is shown at the axes. (**C**) Heatmap of microbial genus-level taxa that contributed to differentiation of sample types and sampling timepoints. Color in the heatmap reflects the relative abundance normalized per taxon, with dark green color indicating higher than average relative abundance and brown color indicating lower than average relative abundance. PCoA: principal coordinate analysis. PERMANOVA: permutational multivariate analysis of variance.

PERMANOVA analysis based on weighted (Figure 1A) and unweighted UniFrac (Figure 1B) distance matrices illustrated remarkable separations among sampling timepoints and sample types, i.e., milk, maternal ileum, and samples of corresponding offspring, with sampling timepoint explaining 7.42% and sample type explaining 31.07% of the total microbiota variation. To differentiate microbial communities between different sample types and sampling timepoints, key contributing bacteria included *Amycolatopsis lactamdurans*, *Paenibacillus amylolyticus*, *Pseudomonas veronii*, *Oceanobacillus profundus*, *Lactobacillus vaginalis,* and *Bacillus safensis* (Figure 1C).

### 3.3. Effect of Maternal MA Additive on the Colonization of Microbiota in Breastmilk and Maternal Ileum

**Figure 2:**
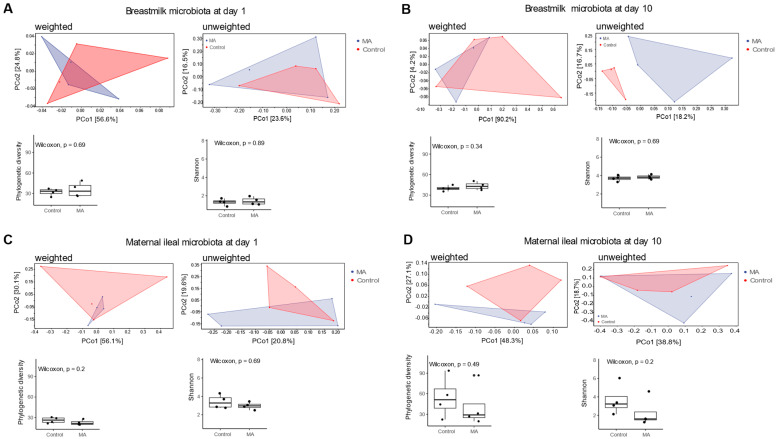
PCoA of microbial composition based on weighted or unweighted UniFrac distance matrices, and microbial richness and diversity in breastmilk at the day of giving birth (**A**) and ten days afterwards (**B**), as well as maternal ileum at the day of giving birth (**C**) and ten days afterwards (**D**). Significant differences between samples based on pairwise weighted and unweighted UniFrac distances at ASV level were tested by PERMANOVA. PCoA: principal coordinate analysis. PERMANOVA: permutational multivariate analysis of variance.

PERMANOVA analysis based on weighted and unweighted UniFrac matrices distance of breastmilk microbiota revealed no significant difference between the breastmilk microbiota of MA-supplemented and control diet-supplemented groups at the day of giving birth (Figure 2A). Nevertheless, ten days after giving birth, the breastmilk microbiota differed remarkably (*p* = 0.066, although not significant) between the MA-supplemented and not-supplemented groups based on unweighted UniFrac distance matric, but not based on weighted UniFrac distance matric (Figure 2B). In terms of maternal ileum microbiota, no significant differences were identified between MA-supplemented and control diet-supplemented groups at the day of giving birth (Figure 2C) and ten days after giving birth (Figure 2D), neither in microbial richness nor diversity.

Collectively, these results indicate that instead of affecting ileal microbiota, supplementation of MA could have a limited impact on breastmilk microbiota. Specifically, later after giving birth, MA additive could affect the breastmilk microbiota by limiting the presence of some minor bacterial groups, but not the presence of dominant bacterial groups.

### 3.4. Effect of Maternal MA Intake on Offspring Intestinal Microbiota

**Figure 3:**
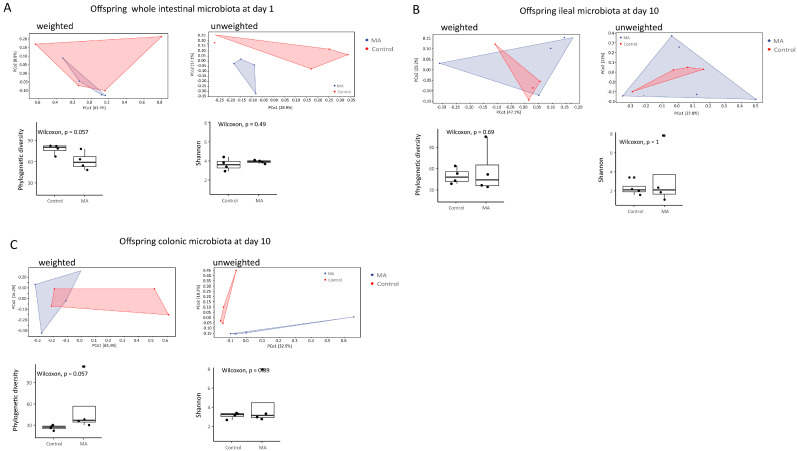
PCoA of microbial composition based on weighted or unweighted UniFrac distance matrices, and microbial richness and diversity in the whole intestine of offspring on the day of birth (**A**) and ten days afterwards in ileum (**B**) and colon (**C**). Significant differences between samples based on pairwise weighted and unweighted UniFrac distances at ASV level were tested by PERMANOVA. PCoA: principal coordinate analysis. PERMANOVA: permutational multivariate analysis of variance.

PERMANOVA analysis based on weighted UniFrac distance matrices revealed no significant difference between the intestinal microbiota of offspring of MA-supplemented mothers and control diet-supplemented mothers (Figure 3A–C). Nevertheless, based on unweighted UniFrac distance matrices, there were significant differences (*p* = 0.035) between the microbiota of these two groups of offspring in the colon ten days after birth (Figure 3C), indicating that the offspring’s colon was the action site of maternal supplemented MA.

### 3.5. Compositional Differences between Treatments and over Time

**Figure 4:**
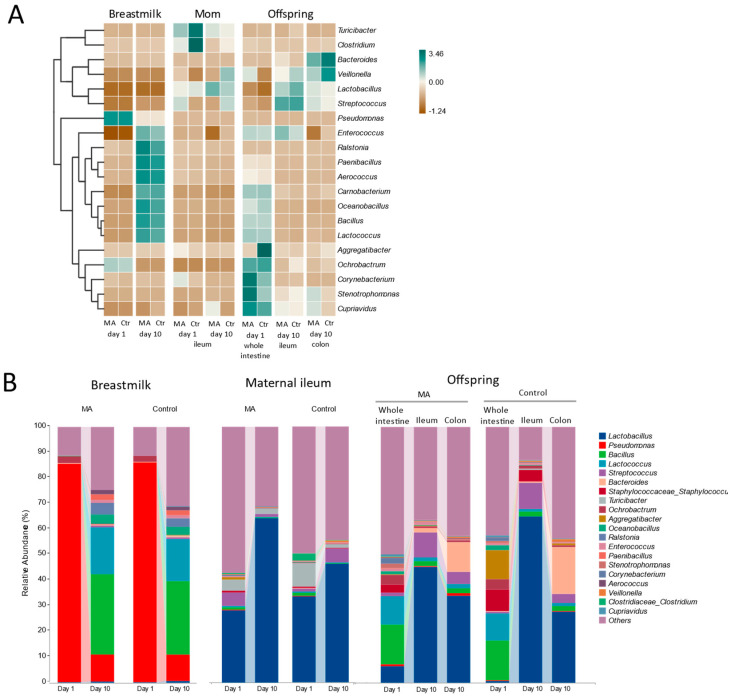
(**A**) Heatmap of top 20 microbial genus-level taxa present in different sample types and sampling timepoints. Color in the heatmap reflects the relative abundance normalized per taxon, with dark green color indicating higher than average relative abundance and brown color indicating lower than average relative abundance. (**B**) Relative abundance of different bacterial genera in different sample types derived from different sampling timepoints. Top 20, ranked based on the average relative abundance across the entire dataset.

**Figure A1:**
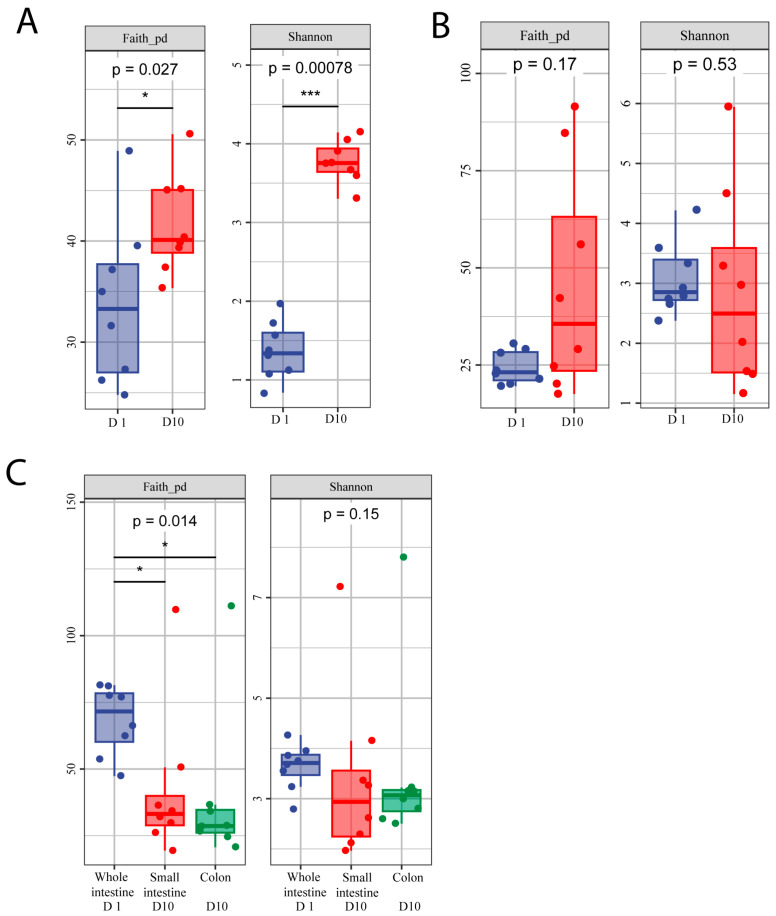
Changes in microbial richness and diversity over time. The microbial richness and diversity of breastmilk (**A**), maternal ileum (**B**) and offspring whole intestine, small intestine and colon (**C**) from the day of giving birth to ten days afterward. *** indicates an extremely significant difference (*p* < 0.001); * indicates a significant difference (*p* < 0.05).

Supplementation of MA did not have a significant impact on the relative abundance of bacterial groups in breastmilk and maternal ileum at the day of giving birth and ten days afterwards (Figure 4A). Nevertheless, from the day of giving birth to ten days afterwards, in addition to the increase in breastmilk microbial diversity (as evaluated using phylogenetic diversity and Shannon index, Figure A1), at genus level, the dominant bacteria changed from *Pseudomonas*, i.e., *Pseudomonas veronii*, at the day of giving birth to *Bacillus* and *Lactococcus* ten days after giving birth (Figure 4B).

Different from breastmilk microbiota, the maternal ileal microbiota and offsprings’ intestinal microbiota ten days after birth were dominated by *Lactobacillus* (Figure 4B). Compared to the offspring of mothers who were supplemented with a control diet, the offspring of mothers supplemented with MA had lower levels of *Aggregatibacter* (i.e., *Aggregatibacter pneumotropica*) on the day of birth and lower levels of *Enterococcus* ten days after birth (Figure 4A). From the day of birth to ten days after birth, there was a notable decrease in the relative abundance of *Bacillus* and *Lactococcus* in the offsprings’ intestinal microbiota, while the relative abundance of *Lactobacillus* increased at the same time. In addition, compared to ileal microbiota, colonic microbiota had a higher relative abundance of *Bacteroides* (i.e., *Bacteroides fragilis*), while ileal microbiota had a higher relative abundance of *Lactobacillus* and *Streptococcus* (Figure 4B).

## 4. Discussion

In the current study, we studied the impact of maternal MA additive on the microbiota of breastmilk, maternal ileum, and the intestinal microbiota of offspring on the day of birth and ten days after birth. We hypothesized that maternal MA additive could affect the microbiota of breastmilk, maternal ileum, and the intestinal microbiota of offspring on the day of birth, as well as ten days after birth. We found that breastmilk microbiota was dominated by *Pseudomonas veronii* at the day of giving birth. Ten days afterwards, the breastmilk microbiota was dominated by *Bacillus* and *Lactococcus*, while microbial diversity had significantly increased. Nevertheless, the additive of MA did not significantly affect the breastmilk microbiota, nor the maternal ileal microbiota, although a remarkable difference in breastmilk microbiota was observed ten days after giving birth. While investigating the influence of maternal MA additive on the offsprings’ intestinal microbiota, we only found a significant difference between the treatment groups based on unweighted UniFrac distance, and this distinction was observed in the colon of the offspring at the ten-day postnatal period.

Consistent with earlier findings that milk microbiota changes along with the lactation stage [38], the current study demonstrated changes in breastmilk microbiota composition and its microbial diversity from the day of giving birth to ten days afterwards. MA additive did not have a significant impact on breastmilk microbiota on the day of giving birth, which contradicts the belief that maternal diet shapes milk microbiota [27]. Thereby, studies were usually based on correlation analysis between dietary food components acquired from questionnaires and breastmilk microbiota [27,39]. Ten days after giving birth, the presence of minor bacterial groups in the breastmilk of MA-supplemented mothers differed remarkably (*p* = 0.066) from control diet-supplemented mothers (Figure 2B). The insignificance between studied groups could in part be attributed to the small sample size of the current study. Altogether, the current study illustrated that maternal MA additive could have a limited impact on breastmilk microbiota, and that the specific impact was more obvious in the presence or absence of certain microbes later after birth.

Zooming in on the microbial composition of breastmilk, the current study found that the dominant rat breastmilk microbiota changed from *Pseudomonas veronii* on the day of giving birth to *Bacillus* and *Lactococcus* ten days afterwards. This finding, however, differed from earlier findings in sow breastmilk, which illustrated the dominance of *Corynebacterium and Streptococcus* as well as the dominance of *Lactobacillus*, two unclassified genera in the families *Ruminococcaceae* and *Lachnospiraceae*, and an unclassified genus in the order *Clostridiales*) [40]. These inconsistencies could in part be attributed to the use of a rat model and to differences in sample collection methods [41] as well as differences in living environments [42]. Nevertheless, species within *Lactococcus* and *Lactobacillus* are generally beneficial bacteria [43].

In line with earlier studies [44], the current study found that different from breastmilk microbiota, maternal ileal microbiota and offsprings’ intestinal (small intestine and colon) microbiota ten days after birth were dominated by *Lactobacillus*. Previous studies showed three *Lactobacillus* strains isolated from the intestines of neonatal piglets alleviated intestinal oxidative stress and protected the intestinal barrier. In addition, no significant impact of MA additive was observed on the microbiota of maternal and offspring ileum, while maternal MA additive significantly affected the colonic microbiota of offspring ten days after birth, highlighting the possibility of modulating infant intestinal microbiota through maternal diet [45]. The current study demonstrated the actual effect of maternal diet on the intestinal microbiota of offspring, contradicting earlier findings based on correlation analysis between maternal diet and offspring microbiota composition [46]. In addition, as the only significant differences in offspring microbiota were observed in the colon, not in the whole intestine nor the ileum, the current study also demonstrated that the action site of (maternal) supplemented MA was not the ileum, but the colon. Specifically, its impact was mainly on the presence of minor bacterial groups, instead of dominant bacterial taxa. Nevertheless, we acknowledge that one limitation of the current study is its lack of microbiota analysis based on maternal colonic content; further studies are needed on the microbes that differ significantly in breastmilk and offsprings’ colons to reveal their antioxidant mechanisms.

Collectively, the current study revealed the effect of maternal MA additive on the microbiota of breastmilk and maternal ileum, as well as the intestinal microbiota of offspring, and illustrated one action site (i.e., colonic microbiota) of maternal MA additive in the offspring. Thus, these findings indicate that maternal MA additive could affect the presence of some microbes in the breastmilk, not immediately on the day of birth, but later (ten days) after birth. These specific microorganisms can be further explored and studied in the future. Moreover, the action site of maternal MA additive was the colon, not the ileum, and its impact was on the presence of minor bacterial groups, not on major bacterial composition. Altogether, the current study will aid in the development of maternal feed for better maternal and offspring health.

## 5. Conclusions

The addition of MA to maternal feed during the perinatal period in rats affected the composition of breastmilk microbiota remarkably (although not significant, *p* = 0.066) ten days after giving birth, specifically regarding the presence or absence of minor microbes. In terms of its impact on the composition of the intestinal microbiota in new-born offspring, the maternal MA additive significantly altered the relative abundance of rare taxa in the offsprings’ colonic microbiota, highlighted the possibility of modulating infant intestinal microbiota through maternal diet. As the postnatal age of the offspring increased, the effect of maternal MA additive on the intestinal microbiota of the offspring became detectable (ten days after birth). Overall, the current study highlighted that maternal MA additive could affect the presence of some microbes in breastmilk ten days after birth, suggested colonic microbiota as a key target affected by maternal MA additive in the offspring, and indicated that MA additive has the potential to relieve oxidative stress by enriching beneficial bacteria. Our conclusions can provide a theoretical basis for the development of antioxidant feed and the improvement of animal welfare in the pig industry.

## Figures and Tables

**Table 1:** Nutrient composition of basic diet.

**Main Ingredient**	**Energy**	**Protein**	**Fats**	**Other**
	corn, wheat	fish meal, chicken meal, yeast powder, soybean meal, alfalfa meal	vegetable oil, soybean oil	amino acids, vitamins, minerals
**Nutritive Index**	**Crude Protein**	**Crude Fat**	**Carbohydrate**	
	21.72%	4.57%	52.96%	

**Table 2:** Production performance of female rats.

Group	Average Weight/g	Average Feed Intake During Pregnancy/g	Average Feed Intake in Lactation/g	Litter Size	Primary Litter Weight/g
Control	311.24 ± 33.32	22.09 ± 1.83	308.90 ± 39.48	13.75 ± 2.45	88.89 ± 14.25
MA	289.12 ± 18.04 ***	17.46 ± 1.55 **	260.05 ± 31.47 *	13,25 ± 2.99	85.40 ± 18.92

*** indicates an extremely significant difference (*p* < 0.001); ** indicates an extremely significant difference (*p* < 0.01); * indicates a significant difference (*p* < 0.05).

## Data Availability

The data presented in this study are available in this article. Further information is available upon request from the corresponding author.
